# A unique heterologous fibrin sealant (HFS) as a candidate biological scaffold for mesenchymal stem cells in osteoporotic rats

**DOI:** 10.1186/s13287-017-0654-7

**Published:** 2017-09-29

**Authors:** Patrícia Rodrigues Orsi, Fernanda Cruz Landim-Alvarenga, Luis Antônio Justulin, Ramon Kaneno, Marjorie de Assis Golim, Daniela Carvalho dos Santos, Camila Fernanda Zorzella Creste, Eunice Oba, Leandro Maia, Benedito Barraviera, Rui Seabra Ferreira

**Affiliations:** 1 Center for the Study of Venoms and Venomous Animals (CEVAP), UNESP – Universidade Estadual Paulista, Botucatu, SP Brazil; 2 Botucatu Medical School, UNESP – Universidade Estadual Paulista, Botucatu, SP Brazil; 3 Botucatu Biosciences Institute, UNESP – Universidade Estadual Paulista, Botucatu, SP Brazil; 4 College of Veterinary Medicine and Animal Husbandry (FMVZ), UNESP – Univ Estadual Paulista, Botucatu, SP Brazil

**Keywords:** Fibrin sealant, Osteoporosis, Cytotoxicity, Snake venom, Fibrinogen

## Abstract

**Background:**

The injection of mesenchymal stem cells (MSCs) directly into the bone of osteoporotic (OP) patients for rapid recovery has been studied worldwide. Scaffolds associated with MSCs are used to maintain and avoid cell loss after application. A unique heterologous fibrin sealant (HFS) derived from snake venom was evaluated for the cytotoxicity of its main components and as a three-dimensional biological scaffold for MSCs to repair a critical femur defect in osteoporotic rats.

**Methods:**

The cytotoxicity of HFS was assessed using a 3-(4,5-dimethyl-2-thiazolyl)-2,5-diphenyl-2H-tetrazoliumbromide (MTT) assay and transmission electron microscopy. The cells were cultured, characterized by flow cytometry and differentiated into the osteogenic lineage. Two-month-old rats underwent ovariectomy to induce OP. After 3 months, a 5 mm critical bone defect was made in the distal end of the rat femurs and filled with HFS; HFS + MSCs; and HFS + MSCs D (differentiated into the osteogenic lineage) to evaluate the effects. An injury control group (injury and no treatment) and blank control group (no injury and no treatment) were also included. The animals were observed at days 14 and 28 by microtomographic (micro-CT) analyses, histologic and biochemical analysis, as well as scanning electron microscopy.

**Results:**

The results revealed that one of the compounds of HFS, the thrombin-like enzyme extracted from snake venom, had no cytotoxic effects on the MSCs. OP was successfully induced, as demonstrated by the significant differences in the levels of 17β-estradiol, Micro-CT analyses and alkaline phosphatase between the ovariectomized (OVX) and non-ovariectomized (NOVX) groups. The histological data revealed that at 14 days after surgery in both the OVX and NOVX animals, the HFS + CTMs and HFS + CTMsD showed a higher formation of bone cells at the site in relation to the control group (without treatment). Collagen formation was evidenced through bone neoformation in all treated and control groups. No morphological differences in the femurs of the NOVX and OVX animals were observed after the surgical procedure. Scanning electron microscopy (SEM) confirmed the histological analysis.

**Conclusions:**

The new HFS composed of two non-toxic components for MSCs showed capacity to promote the recovery of the bone lesions in OVX and NOVX animals at 14 days after surgery. In addition, the HFS enabled the differentiation of MSCs into MSCs D in the group treated with HFS + MSCs. Using the MSCs and/or MSCs D together with this biopharmaceutical could potentially enable significant advances in the treatment of osteoporotic fractures. Future clinical trials will be necessary to confirm these results.

## Background

Clinically, osteoporosis (OP) can manifest as pain, fractures, and physical disability, resulting in independence loss and long-term care. Studies have reported a prevalence of 10% in men – aged between 60 and 79 years old – and 18.5% in women – aged between 40 and 79 years old. In the European Union, there is an estimate of 22 million women and 5.5 million men affected by this illness [1, 2]. According to the World Health Organization (WHO), OP affects approximately 75 million people worldwide [3]. The high incidence commonly occurs in postmenopausal women due to estrogen deficiency, which has a bone antiresorptive action [4]. The fall of this hormone is known as a critical factor impairing cancellous metaphyseal bone and reducing bone mineral density (BMD) [5]. OP is an osteometabolic and multifactorial disease characterized by the loss of bone mass and microarchitecture, leading to increased skeletal fragility and fracture risk complicating the recovery of the long bone-fractures [6–8].

Osteoblasts are sophisticated fibroblasts responsible for bone formation through secretion of the organic components of bone matrix [9, 10]; they indirectly control levels of bone resorption because they regulate the differentiation and activity of the bone-resorbing osteoclasts [10]. Diseases such as OP are associated with altered osteoblast function as a result of a negative balance between the bone-forming activities of osteoblasts and the resorptive activities of osteoclasts [10].

Mesenchymal stem cells (MSCs) are multipotent cells with the capacity to differentiate into other cell types, including osteoblasts, chondrocytes, myocytes, adipocytes, and connective tissue fibroblasts [11]. The use of MSCs to repair tissues has progressively evolved; the goal of cell-mediated therapy is to prolong or replace physiological healing abilities when they are lacking, failing, or progressing too slowly [12]. Use of stem cells in tissue repair has developed progressively and the goal of this therapy is to improve the ability or to replace the restorative capacity of bone tissue when there is partial or complete failure in the repair mechanism [12, 13]. The combination of living cells with a synthetic or natural biomaterial can produce a three-dimensional tissue that is functionally, structurally, and mechanically equal to the original tissue [14]. Different compounds have been used as scaffolds for MSCs. These contribute to repair and regeneration of tissue, and can be classified as both synthetic (hydroxyapatite and tricalcium phosphate) [15] or as biological [16]. Osteoconductive synthetic implants such as hydroxyapatite and tricalcium phosphate have porous structure that facilitates bone growth; however, the lack of osteoinductive potential is a limitation [17].

Although many scaffolds associated with stem cells have been studied in the treatment of bone defects [18, 19], they have been insufficiently researched in the treatment of osteoporotic fractures. Studies of bone mesenchymal stem cells (BMSCs) from osteoporotic patients and animal models have discovered that osteoporosis is often associated with a reduction of BMSCs’ proliferation and osteogenic differentiation [20, 21]. The local injection of normal BMSCs may even improve the bone structure of osteoporotic sites [22]. Enhanced proliferation and differentiation effects of a calcitonin gene-related peptide (CGRP)- and Sr-enriched calcium phosphate cement on bone mesenchymal stem cells and the formation of new bone during osteoporosis-induced bone disorders have been observed [23].

Commercial fibrin sealants (FS) are used as hemostatic, sealant, and adhesive agents to facilitate wound healing in addition to serve as a matrix for the delivery of drugs [24, 25]. These FS are very expensive and composed at least of human thrombin and fibrinogen, which might transmit infectious diseases [26, 27]. The biocompatibility, biodegradability, and cell-binding capacity of FS indicate that they are potentially suitable biological vehicles for use in cell transplantation [25, 28, 29].

A unique heterologous fibrin sealant (HFS) has been studied by our group since the 1990s [28–33]. This sealant is composed by at least two components, i.e., a serine protease extracted from *Crotalus durissus terrificus* venom (a thrombin-like enzyme) and a cryoprecipitate rich in fibrinogen extracted from *Bubalus bubalis* buffaloes’ blood. Animal-derived compounds avoid transmission of infectious diseases from human blood (commercial sealants), and have been tested with success in animals and human beings [34–41].

Fibrin-based biomaterials exhibit several important features of an ideal scaffold, e.g., biocompatibility, biodegradability, and a high affinity to biological surfaces [42]. Scaffolds can provide the necessary support for cells to maintain their specific functions needed to define the shape of new bone [43].

Although many scaffolds associated with stem cells may have been studied in the treatment of bone defects [18, 19], they have not yet been fully studied in the treatment or prevention of osteoporotic fractures. This study aims to investigate the association of HFS with MSCs and MSCs D (differentiated in the osteogenic lineage) in the treatment of bone defects in osteoporotic rats.

## Methods

### HFS scaffold

The HFS was kindly supplied in sufficient quantity for this study by the Center for the Study of Venoms and Venomous Animals at São Paulo State University, Brazil. The components and formula of the applied HFS are contained in its patents (registry number: BR1020140114327 and BR1020140114360). The product is distributed in three vials, stored at -20 °C, and must be mixed and applied immediately at the site of interest [29, 30, 44–46].

### Obtaining the mesenchymal stem cells (MSCs)

Twenty Wistar rats of 10 days age were used as bone marrow donors. The animals used in the research were from the animal house of the Laboratory of Experimental Medicine, from Botucatu, UNESP, São Paulo, Brazil. Extraction of bone marrow cells from donor animals was performed after euthanasia with halothane overdose (CAM > 5%). Bone marrow cells were obtained from the femur by insertion of needle syringe into the bone cavity and then washing with DMEM (Dulbecco's modified Eagle medium, Gibco Laboratories, Grand Island, NY, USA).

### Isolation and expansion of MSCs

After collection of bone marrow stem cells, the pool of material was centrifuged at 2000 RPM for 10 minutes. The obtained material was resuspended in DMEM medium (DMEM; Gibco Laboratories, Grand Island, NY, USA) supplemented with 20% fetal bovine serum (Sigma-Aldrich, St. Louis, MO, USA), 100 μg/ml of penicillin/streptomycin solution (Gibco Laboratories) and 3 μg/ml of amphotericin B (Gibco Laboratories, Grand Island, NY, USA). The cells were plated in culture flasks of 75 cm^2^. The flasks were placed in an incubator with 5% CO_2_ tension at 37 °C. Changes were made to the culture medium every 3 days and cell growth and adherence were monitored by inverted microscope. When cells reached 80% confluence the first cell passage was performed.

To make the passage, the cell culture medium from the flask was discarded and 2 mL of PBS was added for washing, and then Tryple Select (Gibco, Grand Island, NY, USA) for cell trypsinization, and the flask was maintained in an incubator oven for 5 minutes. The flask was removed from the incubator with the cells in suspension. The suspension cells were centrifuged for 10 minutes at 2000 RPM, the supernatant discarded and the pellet resuspended. The cells were counted and used in association with the fibrin sealant for the treatment of the bone defect throughout the experiment.

### MSCs characterization

Surface markers were used to determine the ratio of MSCs and hematopoietic stem cells in the culture after three passages, and the cells were analyzed by flow cytometry (FACSCalibur; BD Pharmingen, San Diego, CA, USA). Positive and negative markers (monoclonal antibodies) for 2 × 10^5^ MSCs included the following: CD73 (purified mouse anti-rat CD73; clone 5 F/B9, BD Pharmingen, San Diego, CA, USA); CD90 (anti-CD90/Thy1-FITC, clone FITC.MRC OX-7; Abcam, Cambridge, MA, USA); CD44 (anti-CD44-PE, clone OX-50; Abcam, Cambridge, MA, USA); ICAM-I (anti-ICAM-I-FITC, clone 1A29; Abcam, Cambridge, MA, USA); RT1 (anti-RT1-Aw2-FITC, clone MRC OX-18; Abcam, Cambridge, MA, USA); CD34 (anti-CD34-PE, clone ICO-115; Abcam, Cambridge, MA,USA); CD11b (anti-CD11b-PE, clone ED8; Abcam, Cambridge, MA, USA); CD45 (anti-CD45-FITC, clone MRC OX-1; Abcam, Cambridge, MA, USA); and MHCII (anti-rat MHC CLASS II RT1D-PE, clone MRC OX-17; Abcam, Cambridge, MA, USA) [38–41]. The isotype controls were secondary antibody anti-mouse IgG, isotype control IgG1-FITC, isotype control IgG1-PE, and MSCs. Analysis was performed using a FACSCalibur™, Flow Cytometry System (BD Biosciences, San Jose, Ca, EUA). The data were analysed using the CellQuest Pro® software. During the cells acquisition were accounted for 20.000 events.

### Osteogenic differentiation of MSCs

We performed only osteogenic differentiation with the intention to use it as one of the treatments in the bone lesion. Previously, these cells were already studied by our research group and differed in the three cell lines – osteogenic, chondrogenic, and adipogenic [16].

When the cell culture reached 70% confluence, the complete culture medium was replaced with a specific StemPro® Osteogenesis Differentiation Kit (Gibco by Life Technologies A10072-01, Carlsbad, CA, USA), which was used with 73% Osteocyte/Chondrocyte Differentiation Basal Medium (Gibco by Life Technologies A10069-01, Carlsbad, CA, USA), 5% Osteogenesis Supplement (Gibco by Life Technologies A10066-01, Carlsbad, CA, USA), 1% penicillin/streptomycin, 1% amphotericin B, and 20% fetal bovine serum (Sigma-Aldrich, St. Louis, MO, USA). The differentiation medium was replaced every 3 days for 12 days. Then, the cells were fixed in ice-cold 70% ethanol, washed in distilled water and stained in 2 mL of alizarin red (Invitrogen Life Science Technologies, Carlsbad, CA, USA) for 30 minutes at room temperature. After the dye was removed, the cells were washed four times in water and observed using an inverted light microscope [16].

### Cytotoxicity assay

The cytotoxicity of the HFS and thrombin-like enzyme (TLE) associated to MSCs were assessed using a 3-(4,5-dimethyl-2-thiazolyl)-2,5-diphenyl-2H-tetrazoliumbromide (MTT) assay (Sigma-Aldrich, Poznań, Poland) [47].

Three concentrations were tested to TLE: 0.5 mg/mL, 0.25 mg/mL and 0.05 mg/mL. Two concentrations were tested with HFS: 100 μL (35.453 mg/mL of calcium chloride; and 6.25 μL (2.21 mg/mL of calcium chloride, 3.25 μg/mL of TLE and 11.25 μg/mL of cryoprecipitate). MSCs were used as a positive control group and culture medium as a negative control. *p* < 0.05 was calculated comparing positive control.

Third-passage MSCs were trypsinized with 2 mL of TrypLE Select (Gibco Laboratories, Grand Island, NY, USA). For the colorimetric test, 5 × 10^4^ cells/mL were used in sterile Eppendorf 96/F-PP microplates with five replicates for each group. High-glucose DMEM (Gibco Laboratories, Grand Island, NY, USA) was added to the wells, and the plate was incubated for 48 hours at 37 °C with 5% CO_2_. After the supernatant was discarded, 100 μL of MTT (1 mg/mL) was added to each well and incubated for 2 hours. The MTT was then replaced with 100 μL/well of dimethyl sulfoxide (DMSO, Thermo Scientific, Waltham, MA, USA). The plates were read at 540 nm in an ELISA reader.

Cell viability was assessed by calculating the arithmetic mean of the quintuplicates and normalized according to the following formula: percentage of cell viability = (absorbance of sample cells - blank absorbance/absorbance of negative control cells - blank absorbance) × 100 [47].

### Transmission electron microscopy (TEM)

To assess the cytotoxicity of HFS associated with the MSCs we used TEM. The HFS and HFS + MSCs were fixed for 3 hours with Karnovsky’s fixative (1% paraformaldehyde, 2.5% glutaraldehyde in 0.1 M phosphate-buffered saline; PBS, pH 7.3). After fixation, the samples were washed with 0.1 PBS, pH 7.3 (3 × 5-minute washes), and then post-fixed in 1% osmium tetroxide diluted in the same buffer for 2 hours. The samples were then washed with distilled water (3 × 10-minute washes), immersed in uranyl acetate (0.5% in distilled water) for 2 hours, dehydrated in increasing concentrations of acetone, and immersed in 100% acetone/Araldite® resin (1:1) for 12 hours. Ultra-thin 90 nm sections were prepared and stained with uranyl acetate (50% in alcohol) for 20 minutes. Subsequently, the sections were stained with lead citrate for 10 minutes. The samples were then analyzed by TEM with an FEI Tecnai Spirit system (Hillsboro, OR, USA).

### Animals and surgical protocols

The animals were handled and the surgical procedures were conducted in accordance with the Brazilian College for Animal Experimentation guidelines, and the protocols were approved by the university’s Ethics Committee in Experimental Animal Use (protocol number 998/2013).

Eighty female Wistar rats (*Rattus norvegicus*) were kept at 21 ± 2 °C under a 12 h light/dark cycle and allowed access to food and water ad libitum. All operations were carried out under sterile conditions with a gentle surgical technique. The surgeon was blinded to the treatment. A single intramuscular dose of fluxinin meglumine (1 mg/kg) was administered after the operation.

### Ovariectomy-induced OP model

To establish an OP model, the rats were subjected to bilateral ovariectomy at 3 months of age, as previously described [8].

### Femur defect model

At 2 months after the ovariectomy, femur defect drilling was performed under general anesthesia, which was induced via the intraperitoneal injection of ketamine and xylazine hydrochloride (1:1) at a dose of 0.10 mL/100 mg. A linear skin incision of approximately 1 cm was made in the distal femoral epiphysis bilaterally, and blunt dissection of the muscles was performed to expose the femoral condyle. After the bone tissue was exposed, a critical defect 5 mm in diameter was created using a micromotor (LB100, Beltec, Araraquara, Brazil), thus promoting bone defects in the distal femoral epiphysis on the right; the lesion was irrigated with saline to prevent thermal necrosis. The treatment was carried out in the defects according to group allocation [48].

Thereafter, rats were divided in two groups: non-ovariectomized (NOVX) and ovariectomized (OVX). Four animals of each group were treated using HFS; HFS + MSCs; HFS + MSCs D. Four animals were injury control (injury and no treatment) and four animals were blank control (no injury and no treatment) to each group. In both groups the animals were euthanized after 14 and 28 days to analyses.

All femurs were removed and analyzed using micro-computed tomography (micro-CT), radiographic and histology.

### Ovariectomy-induced OP model confirmation

Blood samples were collected after 14 and 28 days of the femur defect model surgical procedure and centrifuged to determine the alkaline phosphatase (AP) activity and serum estrogen levels [49]. AP activity was determined using commercially available kits (Bioclin Therapeutics, San Ramon, CA, USA) and an automatic BS-200 analyzer (Mindray Shenzhen, China). The estradiol levels (pg/mL) were assessed via radioimmunoassay using a 1470 Automatic Gama Counter (PerkinElmer, Turku, Finland). All analyzes were performed in duplicate.

### Radiographic evaluation

Radiographic imaging of the rat femurs was conducted on the 14th and 28th days using a digital GE model E7843X system (GE Healthcare, Chicago, IL, USA).

### Microtomographic (micro-CT) analyses

The micro-CT analyses were performed using helical tomography (SCT-7800TC, Shimadzu, Kyoto, Japan). Digitalized data and three-dimensional images of 0.04 cm^2^ of the defective areas were generated by built-in μ-CT software. In these areas, Hounsfield units (HU) were calculated comparing ovariectomized and non-ovariectomized animals.

### Histological processing

After the animals were sacrificed, the femoral bones were removed and fixed in formalin-buffered (4%) PBS for 24 hours, after which the femurs were decalcified in 10% nitric acid for 3 days. The bone samples were dehydrated through a graded series of ethanol (from 70% to 100%) and diaphanized in xylene. To obtain a distinct view of the defect, the orientation and alignment of the femurs were carefully considered during paraffin embedding. Longitudinal serial sections were prepared at a thickness of 6 μm and mounted on histological slides. Hematoxylin and eosin (H&E) staining was used for general histological observations. The sections were evaluated using a Leica DMLB 80 microscope and a Leica DC300FX camera (Bannockburn, IL, USA). The images were analyzed using Qwin software (version 3 for Windows).

### Scanning electron microscopy (SEM)

Bone samples were fixed in 2.5% glutaraldehyde in 0.1 M PBS pH 7.3 for 4 hours. The samples were then removed and washed three times for 5 minutes in distilled water. Subsequently, the samples were immersed for approximately 30 to 40 minutes in 0.5% osmium tetroxide diluted in distilled water, washed in distilled water (3 × 10-minute washes); dehydrated in increasing concentrations of ethanol, dried in a critical point apparatus with liquid carbon dioxide, mounted on appropriate chucks, metallized and gold-coated. The SEM analyses were performed using a Quanta 200 electron microscope (FEI Company, Hillsboro, OR, USA) [16].

### Statistical analysis

All results are expressed as the mean ± standard error, and differences between means were assessed by ANOVA followed by unpaired *t* tests. Discontinuous data were analyzed using X^2^ tests. Differences were accepted as significant at *p* < 0.05. Qualitative observations have been represented following assessments made by two researchers blinded to the experimental designs.

## Results

### MSCs expansion and characterization

**Fig. 1:**
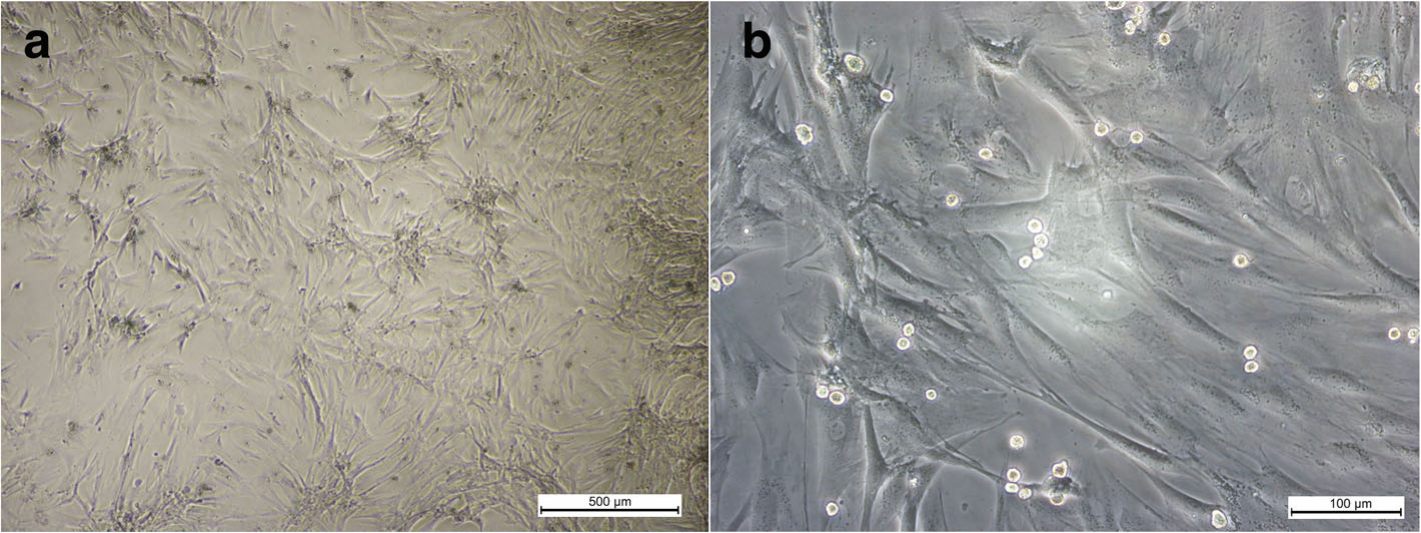
Mesenchymal stem cells (MSCs) culture from bone marrow exhibiting fibroblastoid morphology in the third passage. **a** 500 μm and **b** 100 μm

**Fig. 2:**
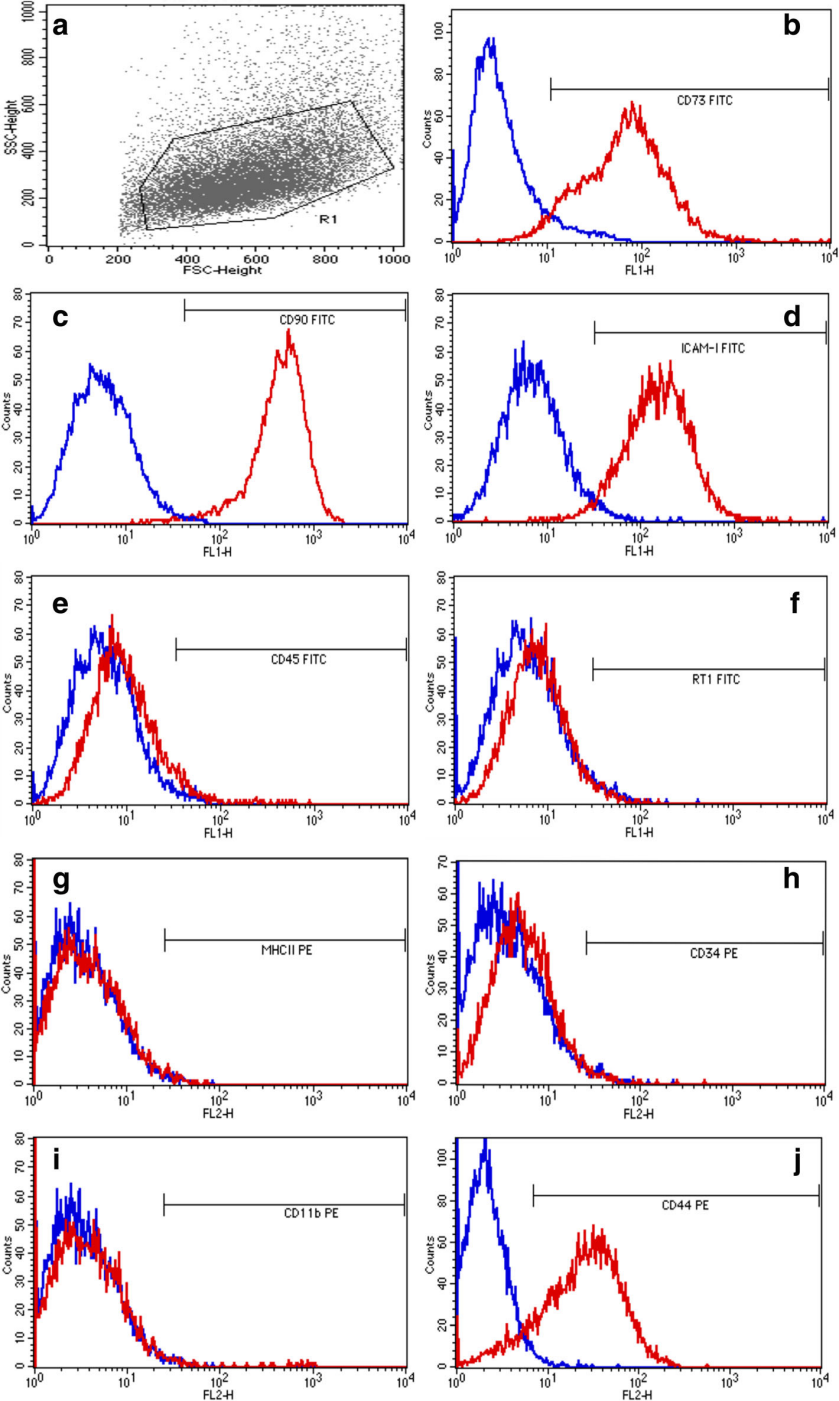
Immunophenotypic analysis of BM-MSCs using the cell surface markers. (**a**) Gate in the cell population; Blue line – Controls (isotype or secundary antibody); Red line – Cell surface markers. Positive: (**b**) CD73-FITC; ( **c**) CD90-FITC; (**d**) ICAM-I-FITC; (**j**) CD44-PE; and negative: (**e**) CD45-FITC; (f) RT1-FITC; (**g**) MHCII-PE; (**h**) CD34-PE; (**i**) CD11b-PE.

MSCs were able to adhere to plastic and exhibited fibroblastoid morphology (Fig. 1). Cells remained in primary culture until reaching 80% confluence after approximately 7 days; they were then subcultured up to the third passage. Flow cytometry indicated that 98.49%, 82.31%, 77.41, and 91.98% of the cells expressed CD90, CD73, CD44, and ICAM-I, respectively (Fig. 2). CD45, CD11b, MHCII, anti-RT1, and CD34 were expressed respectively by 3.53%, 1.74%, 1.45%, 8.46%, and 2.63% of cells (Fig. 2). These results suggested that the cultured cells exhibited the characteristic phenotype of MSCs. Control samples were also used in this study for comparison (Fig. 2).

### Osteogenic differentiation

**Fig. 3:**
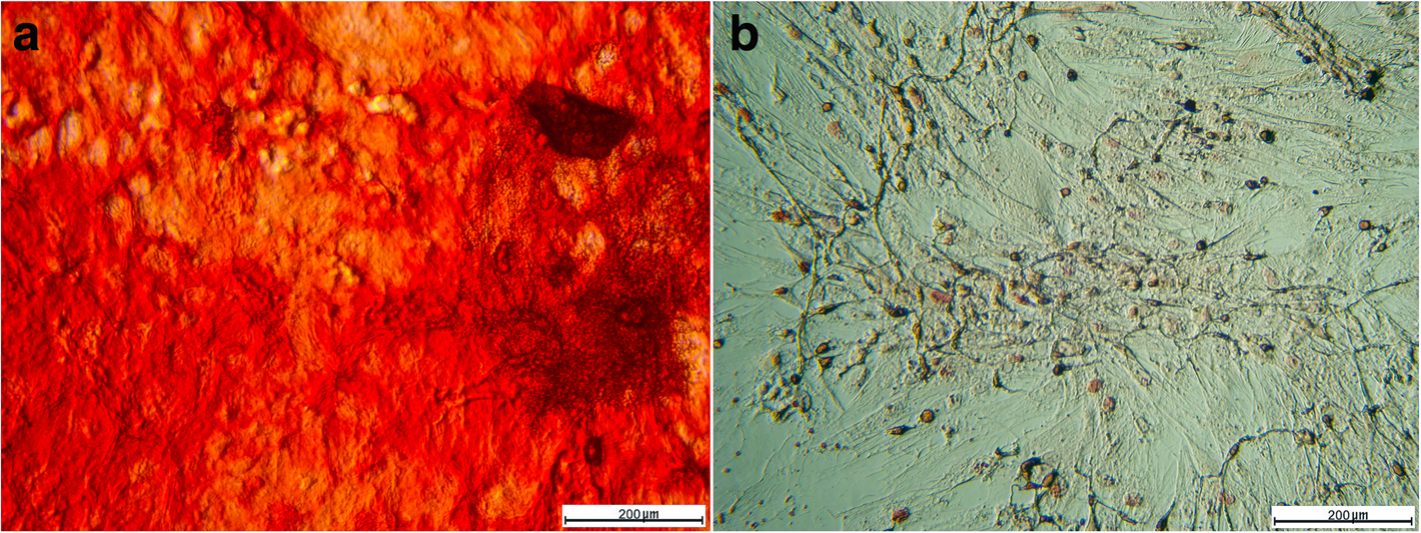
Differentiation assay of MSCs for osteogenic lineage. **a** Positive response to osteogenic differentiation assay. Note the calcium deposits stained with alizarin red. **b** Control assay differentiation cultured in basal medium. *MSCs* mesenchymal stem cells. Bar: 200 μm

Calcium deposits were observed in the MSCs cultures after 12 days of incubation in specific differentiation media. The mineral deposits were detected by the presence of red staining in the extracellular medium, thus confirming the osteogenic differentiation of the MSCs (Fig. 3).

### Cytotoxicity assay

**Fig. 4:**
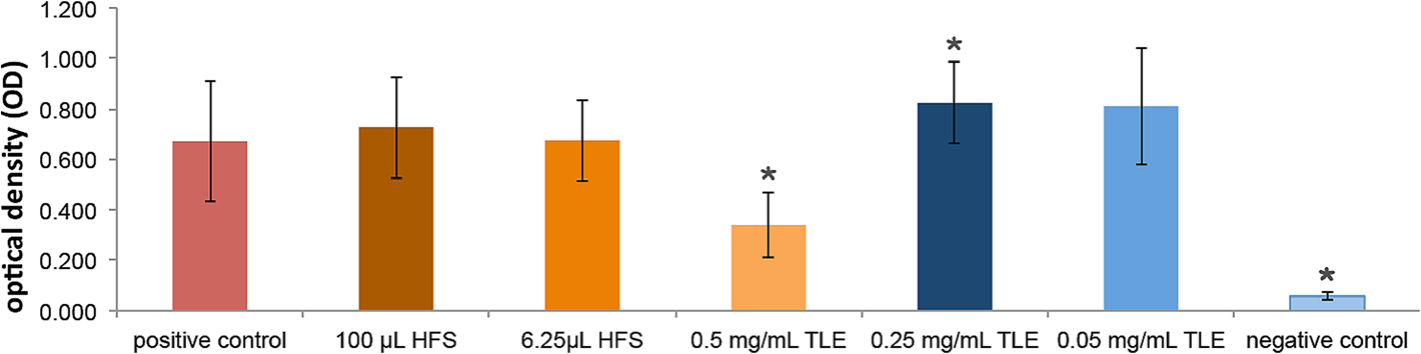
Cytotoxicity assay (MTT) of different concentrations of heterologous fibrin sealant (HFS) and thrombin-like enzyme (TLE). *p < 0.05 means statistical difference in relation to the positive control (cells only)

MTT assay showed that MSCs seeded in concentrations of 0.5; 0.25; and 0.05 mg/mL of the snake venom-derived TLE exhibited 45% (*p* < 0.05), 138% (*p* < 0.05), and 128% of cell growth respectively. The treatments with HFS showed growth of 115% and 108% in the concentrations of 100 μL/mL and 6.25 μL/mL. The higher optical densities (OD) are showed in Fig. 4.

**Fig. 5:**
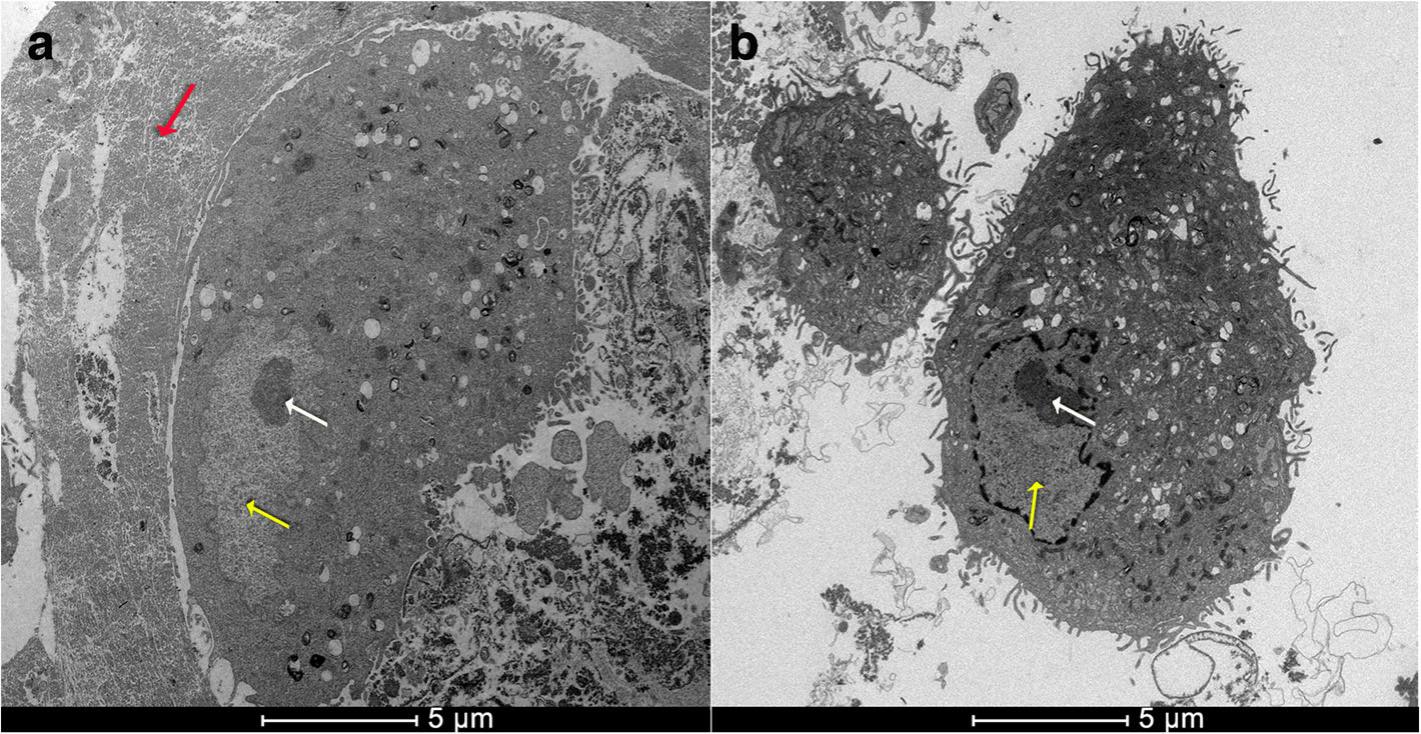
**a** Transmission electron microscopy (TEM) image of mesenchymal stem cells (MSCs) with fibrin sealant (HFS) showed that the HFS was not toxic to cells because the nuclei and cell morphology were undamaged; **b** TEM image of MSCs without HFS. *Red arrow*: HFS; *yellow arrow*: cell nucleus; *white arrow*: nucleolus

TEM analysis showed that the HFS observed around the MSCs was not toxic to cells (Fig. 5). The nuclei and cell morphology were undamaged (Fig. 5a) similarly to the control group (Fig. 5b).

We did not observe cytotoxicity of HFS for MSCs in the two tests performed.

### Serum level of 17β-estradiol

**Fig. 6:**
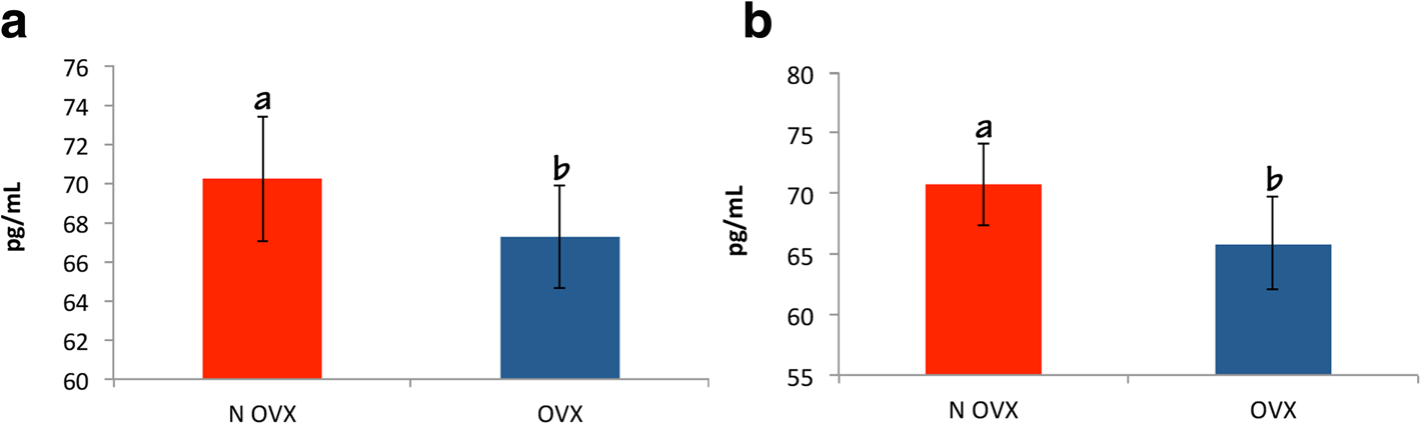
**a** Level of 17β-estradiol at 14 days after femoral surgery (*p* = 0.0155); **b** Level of 17β-estradiol at 28 days after femoral surgery (*p* = 0.0086). The levels of estradiol were lower in the OVX group than in the NOVX group at both time points. Different letters indicate significant differences between the groups (*p* < 0.05). Ovariectomized (OVX) and non-ovariectomized (NOVX) groups

Figure 6 shows estradiol levels in the OVX and NOVX rats at 14 and 28 days after surgery. Levels of estradiol were lower in the OVX group than in the NOVX group at both time points (*p* < 0.05). Thus, the studied period was sufficient to cause a decrease in estradiol and mimic OP in the OVX rats.

### AP activity

AP activity was quantified after 14 and 28 days of induction of lesion femur. The AP activity was significantly higher (*p* < 0.05) in the OVX group than in the NOVX group to both time points (Table 1).

**Table 1:** Dosage of alkaline phosphatase (U/L) levels in the serum of female rats (n = 20/group) at 14 and 28 days after femoral surgery

Period	Groups	Alkaline phosphatase (U/L)
14 days	Ovariectomized	157.31 ± 14.59^a^
	Non-ovariectomized	122.13 ± 8.83^b^
28 days	Ovariectomized	181.75 ± 9.16^a^
	Non-ovariectomized	134.59 ± 8.25^b^

Means ± SEM (*p* < 0.05)

^a,^Statistically significant differences between groups in the period of 14 days (*p* = 0.0405)

^b^Statistically significant differences between groups in the period of 28 days (*p* = 0.0007)

### Radiographic data

**Fig. 7:**
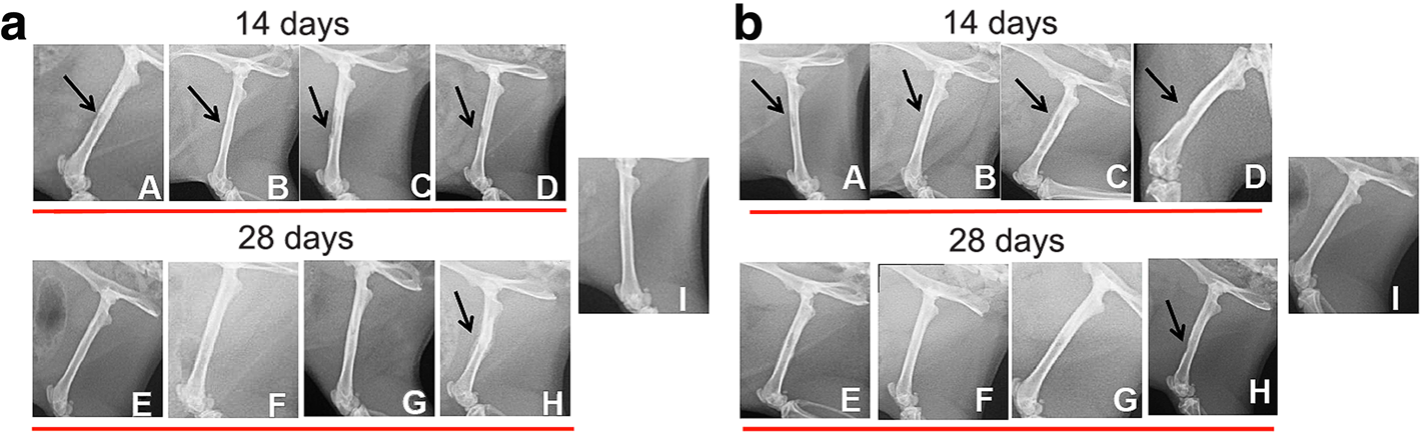
**a** Radiographic images at 14 (A-D) and 28 days (E-H) after femoral surgery in non-ovariectomized rats (NOVX). **b** Radiographic images at 14 (A-D) and 28 days (E-H) after femoral surgery in ovariectomized rats (OVX). A and E (heterologous fibrin sealant treatment); B and F (fibrin sealant (HFS) + mesenchymal stem cells (MSCs) treatment); C and G (HFS + mesenchymal stem cells differentiated into the osteogenic lineage (MSCs D) treatment); D and H (injury, control); I (non-injury, control); *Black arrow*: injury

On day 14 we observed the bone lesions in all animals of groups, non-ovariectomized group (NOVX) and ovariectomized group (OVX). On the 28th day, we observed recovery of the lesion in animals of both treated groups (HFS, HFS + MSCs, HFS + MSCs D). The X-ray evaluation on the 28th day showed that the treatments were able of helping in bone recovery when compared to the control group. In the control group that received no treatment we observed the formation of a bone callus at the site of the lesion (Fig. 7).

### Micro-CT data

Hounsfield scale was used in micro-CT to verify the femur bone density of OVX and NOVX rats. Statistical analysis revealed the difference (*p* < 0.05) in bone density between the groups OVX (930.74) and NOVX (1031.30) at 28 days.

### Histological evaluation

**Fig. 8:**
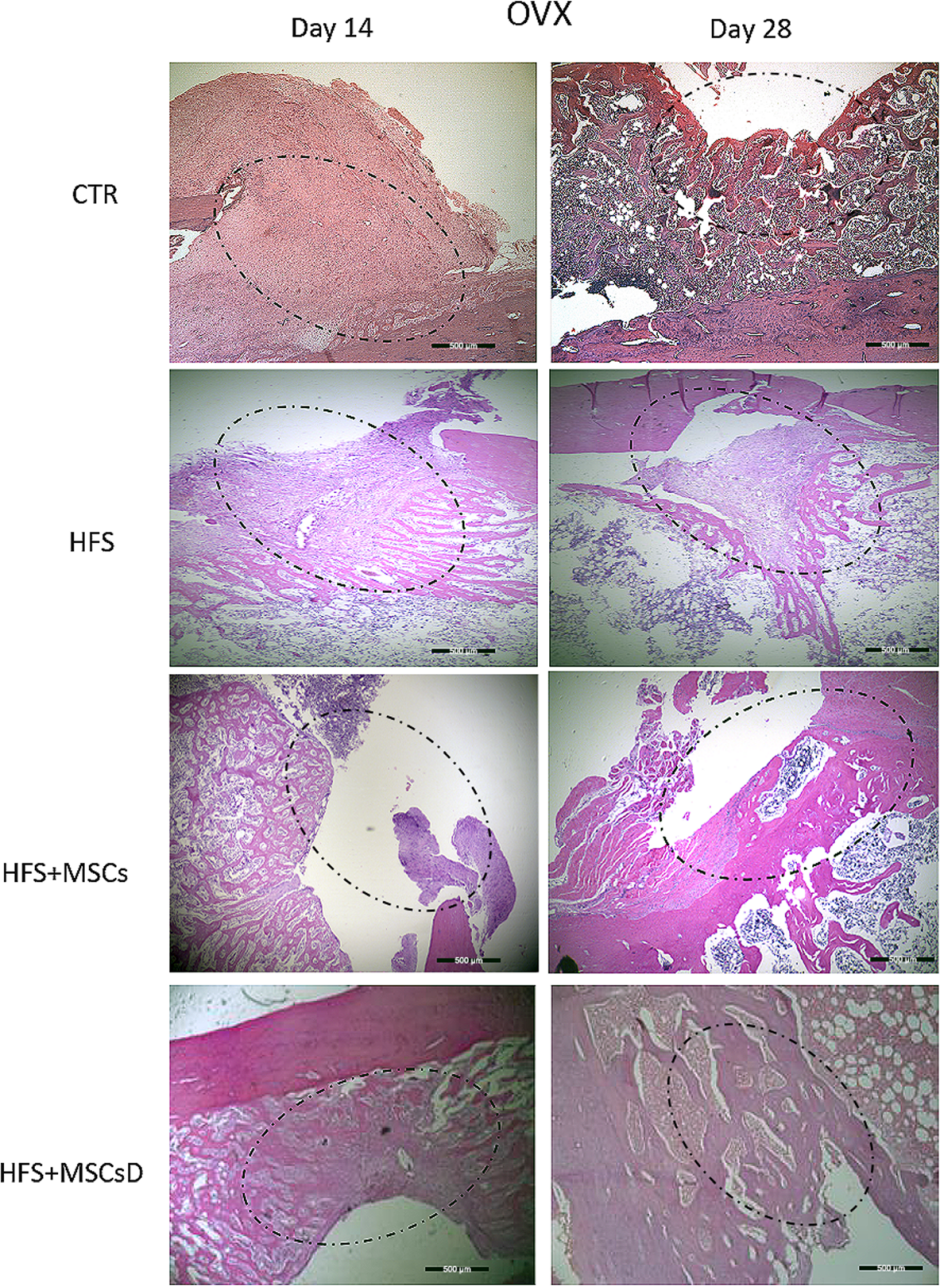
Histological results comparing bone lesions of OVX rat femurs at 14 and 28 days after femoral surgery. CTR: (injury, control); heterologous fibrin sealant (HFS); heterologous fibrin sealant (HFS) + mesenchymal stem cells (MSCs); HFS + mesenchymal stem cells differentiated into the osteogenic lineage (MSCs D). *Dotted circle*: bone injury (hematoxylin and eosin). Bar: 500 μm, magnification: ×4

Histological analyses stained with hematoxylin and eosin (H&E) of the femoral lesion of ovariectomized (OVX) rats showed that treatments of SF + CTMs differentiated or not differentiated in osteogenic lineage presented bone cell formation at the lesion site compared to the control group on 14th day (Fig. 8). At 28 days, we observed that the repair of the bone lesion was progressive, including in the control group (Fig. 8).

**Fig. 9:**
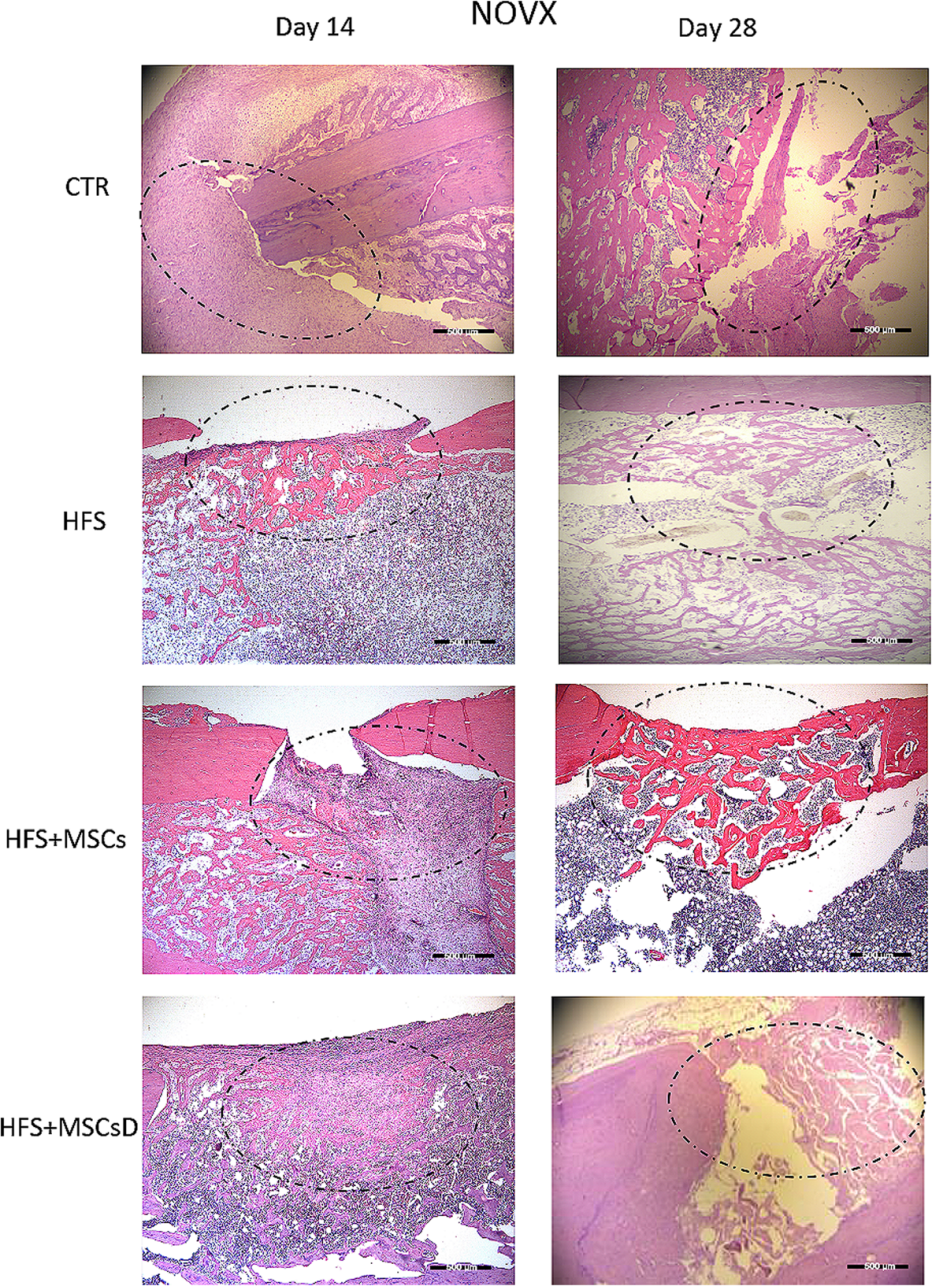
Histological results comparing bone lesions of NOVX rat femurs at 14 and 28 days after femoral surgery. CTR: (injury, control); heterologous fibrin sealant (HFS); heterologous fibrin sealant (HFS) + mesenchymal stem cells (MSCs); HFS + mesenchymal stem cells differentiated into the osteogenic lineage (MSCs D). *Dotted circle*: bone injury (hematoxylin and eosin). Bar: 500 μm, magnification: ×4

Non-ovariectomized animals (NOVX) showed a similar result at both 14 and 28 days (Fig. 9). At 14 days, it was possible to observe the beginning of bone cell formation at the lesion site, which was not observed in the control animals (Fig. 9). In the ovariectomized animals (OVX), at 28 days, it was possible to observe possible recovery progression in the lesion, as well as in the control animals (Fig. 9).

### Scanning electron microscopy (SEM)

**Fig. 10:**
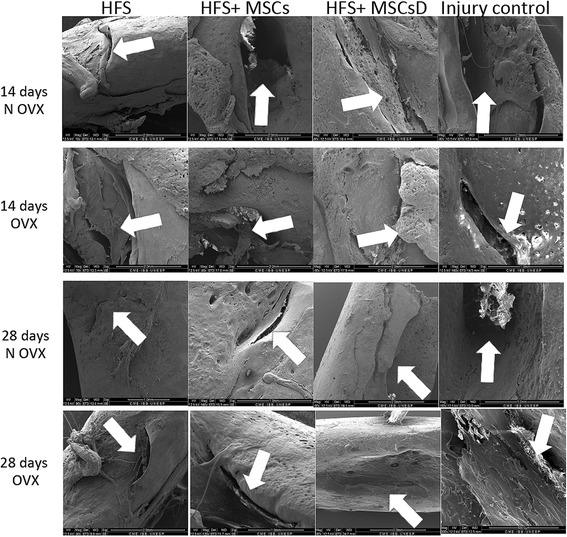
Scanning electron microscopy images of OVX and NOVX rat femurs at 14 and 28 days after femoral surgery. *White arrow*: injury site. Pictures of the MEV are in different magnitudes

SEM analysis showed the bone structure at the lesion site. The bone lesion was observed at 14 days after surgery in the femur of rats in both the NOVX and OVX groups (Fig. 10).

At 28 days after the femoral surgery in OVX and NOVX rats, reduction of the bone lesion after a critical defect was observed. The groups treated with HFS, HFS + MSCs and HFS + MSCs D presented a reduction of the bone lesion compared to the control group (no treatment) (Fig. 10). Bone structure recovery was similar in both the OVX and NOVX groups at both time points.

## Discussion

According to the literature, when a three-dimensional biomaterial is designed to be an ideal scaffold (three-dimensional) it is necessary: to provide a spatially correct position of cell location; promote cell-biomaterial interactions, cell adhesion, and extracellular matrix deposition; to permit sufficient transport of gases, nutrients, and regulatory factors to allow cell survival, proliferation, and differentiation; to biodegrade at a controllable rate that approximates the rate of tissue regeneration; and to provoke a minimal degree of inflammation or toxicity in vivo [50, 51]. The commercial-natural fibrin scaffolds consist of cross-linked fibrin network biomaterial, meet most of these criteria, and provide a structure similar to the native extracellular matrix [52, 53]. Recently [16], it was discovered for the first time that the HFS besides being cheaper than commercial scaffolds and not transmitting infectious diseases by human blood, especially viruses, can be used in vitro as a scaffold for MSCs. In this study, we are searching to see whether the HFS is a good in vivo scaffold for transplantation of MSCs to osteoporotic bone tissue-rats too.

This new HFS was evaluated for the first time as a biological scaffold for MSCs in the repair of critical femur defects in osteoporotic rats. Adult MSCs can be obtained through simple techniques and are capable of differentiating into adipocytes, chondrocytes, and osteogenic precursor cells [12].

MSCs were characterized by confirming that the surface markers CD73, CD90, ICAM-I, and CD44 were expressed and that CD34, CD45, MHCII, and CD11b were not expressed. In the present study, the MSCs expressed CD90 (98.4%), CD73 (82.31%), CD44 (77.41%), and ICAM-I (91.98%) and did not express CD45 (3.53%), CD11b (1.74%), MHCII (1.45%), RT1 (8.46%), or CD34 (2.63%), which was in agreement with the results of several studies [54–57]. The MSCs underwent osteogenic differentiation after 12 days of incubation in specific differentiation medium according to previous results [16].

Commercially available sealants consisting of human fibrinogen and thrombin are not toxic to and can serve as excellent scaffolds for MSCs [16, 58]. One of component of the new HFS is a serine protease purified from *Crotalus durissus terrificus* venom with thrombin-like enzyme activity. As it is a foreign protein to warm-blooded animals, the cytotoxicity of this component against the MSCs was evaluated in vitro for the first time. The serine protease was assessed individually and was non-toxic to the MSCs at any of the measured concentrations, as confirmed by the cell viability (%) results. Surprisingly, this serine protease also showed the ability to stimulate cell proliferation and resulted in higher optical density (OD) values than those of the control group. After mixing both components (serine protease + cryoprecipitate + calcium chloride) the OD could not be assessed due to the intensely gelatinous consistency. Analysis by transmission electron microscope (TEM) showed that the HFS was not toxic to cells, because the nucleus and cell morphology showed full, very similar to the control group. The HFS involved the MSCs and showed a scaffold for cells. The HFS is not toxic to the cells and can be a potential candidate as a scaffold to MSCs.

The OP induction period in rats is variable and can require between 7 weeks and 3 months, provided the rats undergo ovariectomy by the time they are 3 months old [8, 59–62]. In this study, ovariectomy was performed in 3-month-old rats, which were then used for the surgical procedure on the femur 2 months later. Various parameters were used to validate this experimental model, including serum levels of estradiol [49], bone density as assessed by micro-CT [63–66], and AP activity after the defects were introduced [49].

Lower estradiol levels in the OVX rats compared to those in the NOVX rats showed that the period used was sufficient to induce OP, which was in agreement with the previous findings of several authors [8, 59–62]. Estradiol is an estrogen hormone that is involved in the induction of OP when present below the recommended levels [67]. The evaluating of bone density was made by micro-CT analysis, where statistical analyses revealed significant differences in bone density between the groups ovariectomized (930.74) and non-ovariectomized (1031.30). OP has a deleterious effect on the biological repair of fractures. Experiments conducted in this area often aim to improve physiological condition and cellular performance, particularly in metabolic and degenerative diseases [68].

Low levels of AP activity indicate reduced bone formation and bone mass, which also decrease with age; however, high levels can be observed in the first few weeks after surgery or a fracture, especially after long bone fractures [69, 70]. In this study, the observed AP activity was significantly higher in OVX rats than in NOVX rats at both time points. In our research, despite the bone mineral density, estradiol levels, and AP activity presenting a difference between groups OVX and NOVX the bone morphologic parameters did not present a difference.

Some researchers have successfully reversed OP by the intravenous transplantation of MSCs [71, 72]. Others have observed significant improvement in osteoporotic bone structure with the injection of MSCs at the site of injury [22]. However, injecting MSCs peripherally to treat bone defects can lead to the reabsorption and loss of the MSCs into the bloodstream, reducing their level in the targeted area. In this study, we aimed to confine and maintain the MSCs at the injury site by using the HFS together MSCs. Fibrin derivatives are widely used in tissue engineering applications [73], which allow them to serve as an appropriate scaffold for delivery, fixation and cell proliferation [16, 32, 33].

Through the histological analyses we can observe that at 14 days after surgery, in both OVX and NOVX groups, the animals treated with HFS + MSCs and HFS + MSCsD showed a higher formation of bone cells at the injury site in relation to the control, suggesting that the sealant served as a scaffold and allowed the cells to remain at the targeted site. MSCs have been shown to promote bone repair when they are associated with a biological scaffold [74, 75]. We did not observe this qualitative difference at 28 days when compared to the control group. The results of the histological analysis indicated that both treated groups as well as the control group there was formation of collagen evidenced bone neoformation.

Through the SEM analysis, we can observe that at 14 days after surgery in the femur of the rats, all the treated groups presented bone lesion. On the 28th day, the lesions remained visible in all groups, however, we can observe recovery of the lesion over the period; however, it was not possible to observe total bone repair. A period of 6 weeks is not long enough for the complete recovery of a critical defect in a rat femur [48].

## Conclusions

In this study, we evaluated the effect of HFS associated with both MSCs and MSCs D on a rat femur defect model. Overall findings of this study suggest that this association promotes a greater formation of bone cells at the site in relation to the control group at 14 days, in both OVX and NOVX animals. In addition, the HFS enabled the differentiation of MSCs into MSCs D in the group treated with HFS + MSCs. HFS should be further investigated for their mechanical properties to be clinically applicable and acceptable in functional tissue engineering approaches as a scaffold by stem cells. In conjunction with MSCs and/or MSCs D, this biopharmaceutical could potentially enable significant advances in the treatment of osteoporotic fractures.

## Abbreviations

AP: Alkaline phosphatase
BMSCs: bone mesenchymal stem cells
FS: fibrin sealants
HFS: heterologous fibrin sealant
MSCs D: Differentiated into the osteogenic lineage
MSCs: Mesenchymal stem cells
MTT: 3-(4,5-dimethyl-2-thiazolyl)-2,5-diphenyl-2H-tetrazoliumbromide
NOVX: Not ovariectomized
OP: osteoporosis
OVX: Ovariectomized
SEM: Scanning electron microscopy
TEM: Transmission electron microscopy
TLE: thrombin-like enzyme
